# USE OF RWD TO GENERATE RWE IN THE ALZHEIMER'S DISEASE SETTING: CHALLENGES AND FUTURE DIRECTIONS

**DOI:** 10.1093/geroni/igac059.773

**Published:** 2022-12-20

**Authors:** Julia DiBello

**Affiliations:** Merck & Co., Inc., West Point, Pennsylvania, United States

## Abstract

There is considerable interest by many stakeholder groups in developing new therapies in Alzheimer’s disease (AD). Increasing interest in the treatment of pre-clinical disease and measurement of associated long term outcomes using RWD is being explored as the use of RCTs in this context can be limited. Many challenges exist in generating RWE to support regulatory submissions in this disease population including lack of important clinical variables such as disease severity and progression in existing data sources. We will discuss the AD RWD landscape with a focus on challenges and future directions for the use of RWD to generate RWE supportive of regulatory submissions for pharmaceutical products.

